# Clinical Expression of Familial Hypercholesterolemia in Patients from France and French Canada Carrying Identical-by-Descent Pathogenic *LDLR* Gene Variants: A Proof-of-Concept Study

**DOI:** 10.3390/jcm13195725

**Published:** 2024-09-26

**Authors:** Miriam Larouche, Olivier Bluteau, Alain Carrié, Alex Lauzière, Etienne Khoury, Diane Brisson, Daniel Gaudet, Antonio Gallo

**Affiliations:** 1 Departement of Medicine, Université de Montréal and ECOGENE-21, Chicoutimi, QC G7H 7K9, Canada; miriam.larouche@ecogene21.org (M.L.); alex.lauziere@usherbrooke.ca (A.L.); etienne.khoury@umontreal.ca (E.K.); diane.brisson@ecogene21.org (D.B.); 2 Sorbonne-Université, Assistance Publique—Hôpitaux de Paris (APHP), INSERM Unité de Recherche sur les Maladies Cardiovasculaires et Métaboliques (UMRS) 1166, Hôpital Pitié-Salpêtrière, 75013 Paris, France; olivier.bluteau@aphp.fr (O.B.); alain.carrie@aphp.fr (A.C.); antonio.gallo@aphp.fr (A.G.)

**Keywords:** familial hypercholesterolemia, founder effect, identical-by-descent variant

## Abstract

**Background:** Studying patients carrying identical-by-descent (IBD) pathogenic gene variants allows us to control for the disease-causing genetic background and to more accurately document the impact of modifiers. Familial hypercholesterolemia (FH) is characterized by elevated low-density lipoprotein cholesterol (LDL-c) levels and premature atherosclerosis and is often caused by defects in the *LDLR* gene. There is a high prevalence of FH in French Canada as a result of a founder effect from France in the 17th century. Several FH patients currently living in French Canada (founder population) and in France (colonizing population) carry IBD FH-causing variants. The expression of FH is affected by environmental and genetic modifiers, and patients with IBD variants may present different characteristics. **Methods:** In this study, we compared FH clinical expression patients carrying IBD *LDLR* pathogenic variants living in France or Canada. Four IBD variants, namely c.259T>G p.(Trp87Gly), c.2000G>A p.(Cys667Tyr), c.682G>A p.(Glu228Lys), and c.1048C>T p.(Arg350*), were selected. Untreated plasma lipid profiles, the apolipoprotein E (APOE) genotype, cardiovascular risk factors, and the occurrence of symptomatic ASCVD were compared in 105 adult carriers (30 from France and 75 from French Canada). **Results:** All parameters were similar between the two populations, except for untreated total cholesterol (10.14 ± 1.89 mmol/L vs. 8.65 ± 1.84 mmol/L, *p* = 0.0006) and LDL-c concentrations (7.94 ± 1.86 mmol/L vs. 6.93 ± 1.78 mmol/L, *p* = 0.016), which were significantly higher in FH patients living in France, an observation that was revealed across all studied *LDLR* variants. **Conclusions:** This study illustrates that FH patients sharing IBD pathogenic *LDLR* variants that have evolved in different geographic, cultural, and socio-economic environments for hundreds of years differ in terms of cholesterol levels, highlighting the importance of better understanding the interplay between genetic and environmental modulators of FH expression.

## 1. Introduction

Familial hypercholesterolemia (FH) is characterized by the accumulation of low-density lipoprotein (LDL) particles and elevated LDL-cholesterol (LDL-c) levels due to the lack of functionality or availability of LDL receptors (LDLR). FH is most often caused by pathogenic LDLR gene variants or by variants in genes having deleterious effects on LDLR functions [1,2]. Patients affected by FH generally have elevated LDL-c levels from birth (above 5 mmol/L in adulthood) and are at risk of developing atherosclerosis early in life [3,4]. FH prevalence is estimated at 1:250 globally, although its prevalence is significantly higher in some isolates or founder populations [5].

FH expression is affected by multiple modifiers and varies from one individual to another, even among carriers of the same FH-causing pathogenic variant [4]. Environmental and genetic modifiers can directly affect LDL-c concentration, and an increasing amount of evidence shows that these modifiers can also impact the epigenome and modulate FH clinical expression [6,7]. Unlimited combinations of genes and environmental factors therefore contribute to each person’s unique blend of traits, health, and identity, making it difficult to precisely predict the individual trajectory of FH expression and response to treatment.

Identical-by-descent (IBD) pathogenic gene variants have emerged as interesting candidates to study the genetic underpinnings of atherosclerotic cardiovascular disease (ASCVD) as they are shared by individuals via common ancestors. Studying patients with IBD gene variants allows us to control for the disease-causing genetic background and to more accurately document the impact of modifiers (genetic, clinical, environmental, or socio-economic). Many research efforts have indeed been supporting the idea that studying IBD segments consists of a powerful method for controlling genetic background, while providing a more nuanced understanding of the role of various modifiers in disease expression and susceptibility. Additionally, advances in IBD detection methods have been recently developed, such as the RaPID tool, which aims to improve the ability to identify segments with high accuracy and speed, despite having large biobank-scale databases. In addition to controlling the genetic background, these tools are helpful for investigating the impact of environmental and other modifiers on disease expression, while detecting population structure and familial relationships in specific therapeutic areas [8,9].

**Figure 1:**
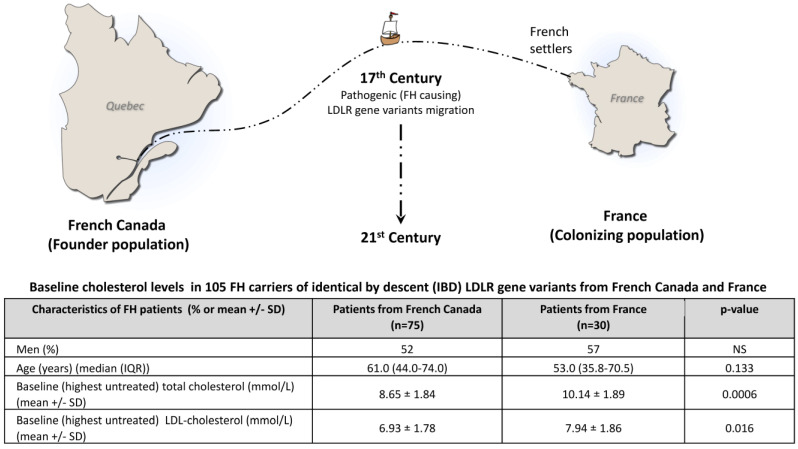
Baseline (untreated) total cholesterol and LDL-cholesterol levels in FH carriers of identical-by-descent (IBD) pathogenic *LDLR* gene variants in 75 FH patients from the French-Canadian founder population and 30 patients from France (colonizing population). Patients from both countries carried either the c.259T>G p.(Trp87Gly) rs121908025, c.2000G>A p.(Cys667Tyr) rs28942083, c.682G>A p.(Glu228Lys) rs121908029, or c.1048C>T p.(Arg350*) rs769737896 *LDLR* gene variants, all pathogenic, having a proven French founder effect and having evolved in a different environment for 4 centuries. Both groups were comparable for ApoE genotype, smoking habits, anthropometric measurements, diabetes, and other cardiovascular risk factors. Patients were matched for age and sex (≥3:1 for the p.(Trp87Gly) variant). Baseline cholesterol levels were significantly lower among French-Canadians in all genotypes (see text).

French-Canadians provide an interesting example of a founder population where documented IBD pathogenic LDLR variants are more prevalent and originate from a common ancestral source from France in the 17th century (Figure 1) [10]. Consequently, some French and French-Canadian patients, today living on different continents, carry the same IBD FH-causing gene variants that have evolved for 4 centuries in different environments. In this proof-of-concept (PoC) study, we compared LDL-c levels and other clinical characteristics in heterozygous FH (HeFH) patients from France and French Canada carrying IBD gene variants. 

## 2. Methods

Four IBD FH-causing *LDLR* variants, namely c.259T>G p.(Trp87Gly) rs121908025, c.2000G>A p.(Cys667Tyr) rs28942083, c.682G>A p.(Glu228Lys) rs121908029, and c.1048C>T p.(Arg350*) rs769737896 originating from France with a proven French-Canadian founder effect were selected [10,11]. During their initial visit to the lipid clinics, FH patients were assessed by a multidisciplinary team. Data on lipid profiles, FH-related genotypes, treatment regimen, metabolic syndrome-associated parameters, and cardiovascular risks were collected from those who consented to participate in this study. Plasma lipid profiles at baseline (untreated), ApoE genotype, cardiovascular risk factors, and the occurrence of symptomatic ASCVD were compared in 105 adult carriers (30 from France and 75 from French Canada). For the most frequent variant, p.(Trp87Gly), patients in both groups were matched for age (±1 year) and sex in a ≥3:1 ratio. For the other variants, matching was dependent on the number of subjects. Data comparisons were made using Chi-square, Fisher’s exact test, Student’s *t*-tests and Wilcoxon–Mann–Whitney for independent samples. Statistical analyses were performed using SPSS package version 25 (IBM Corp., Armonk, NY, USA).

This research study (French National Agency for the Safety of Medicines and Health Products, protocol reference 2014-A01549–38 and its Canadian counterpart, protocol reference ECO HyperTG-Hyperchol) were conducted in accordance with the principles of the Declaration of Helsinki, consistent with the Good Clinical Practice guidelines of the International Conference on Harmonization. Ethical approval was obtained in France from the CCTIRS (Comité Consultatif sur le Traitement de l’Information en matière de Recherche dans le domaine de la Santé) and the CNIL (Commission National de l’Informatique et des libertés) in 2015 and in Canada from Advarra IRB in 2014. All subjects were screened at their respective lipid clinics and agreed to participate in this study. Informed consent was obtained from all participants, and a code that systematically de-identifies all clinical data was assigned to each subject [12]. Participants were included in this analysis based on the availability of lipid-associated parameter data.

## 3. Results

Subjects from both populations were comparable in terms of sex, age, smoking status (current or not), statin intolerance, diabetes, obesity, and hypertension (Table 1). Although not statistically significant, the prevalence of ApoE4 in this study was almost 2-fold more elevated among French-Canadians (29% vs. 17%). Mean baseline untreated total cholesterol (10.14 ± 1.89 mmol/L vs. 8.65 ± 1.84 mmol/L, *p* = 0.0006) and LDL-c concentrations (7.94 ± 1.86 mmol/L vs. 6.93 ± 1.78 mmol/L, *p* = 0.016) were significantly higher in the French cohort (Figure 1), and this was observed across all FH-causing variants (Table 2). The coronary artery anatomy of those who had a coronarography will be the subject of another publication.

## 4. Discussion

In this PoC study, we used LDL-cholesterol as a probe to highlight differences in HeFH expression between patients from two populations sharing IBD *LDLR* pathogenic variants. It illustrates that FH patients of similar age, sex, and risk profile, originating from France and French Canada, sharing well-documented IBD pathogenic LDLR variants having evolved in different geographic, cultural, and socio-economic environments for hundreds of years, may differ in terms of cholesterol levels (Figure 1), highlighting the importance of better understanding the interplay between genetic and environmental modulators of FH expression.

Higher plasma total cholesterol and LDL-c concentrations were observed in FH patients from France despite the fact that the ApoE4 allele (generally associated with higher cholesterol concentration) was 2-fold more prevalent among French-Canadians, who presented lower LDL-c than their French IBD FH counterparts.

Since the colonization of New France in the 17th century, the dissemination of FH-causing gene variants in French Canada was important due to large pedigrees (often > 12 children per nuclear family) and limited population migration movements due to the harshness of transport routes [11]. The combination of these factors led to a phenomenon called endogamy, resulting in the high prevalence of IBD FH in French Canada (1:80), which is much higher than in France and the rest of the world (1:250) [11]. In addition, most cases of FH in French Canada were mainly caused by a small number of IBD *LDLR* pathogenic variants [11]. The historical high prevalence of FH in French Canada is not a consequence of consanguinity but of endogamy [13]. Comparing the expression of IBD genetic diseases in a founder population and in the colonizing population offers a unique opportunity to explain differences in the clinical expression or the response to interventions, controlling for the effect of the disease-causing variant. Indeed, IBD pathogenic variants that have evolved for hundreds of years in different geographic environments allow us to control for the variance in FH clinical expression that is specifically due to the pathogenic variant, which is the case of LDL-c, the main feature of FH. One advantage of IBD pathogenic variants is that differences in the clinical expression of the disease are not determined by the pathogenic variant itself but rather by other modifying factors. This constitutes a real advantage in identifying key genetic, epigenetic, or environmental factors affecting FH expression, risk trajectory, or response to treatment. Specifically, when controlling for the genetic cause of FH, observed differences in LDL-c could contribute to the identification of genetic variants that increase susceptibility to LDL accumulation and atherogenicity beyond *LDLR*.

It is well documented that other genes, environmental factors, nutritional habits, physical activity, bacterial exposure, stress, and other factors affect the epigenome, gene expression, and the microbiota and could highly impact the lipid profile or ASCVD risk [14]. By controlling for the FH-causing gene variant (using IBD variants), the analysis of modifiers is simplified, potentially uncovering novel pathways that influence FH expression, risk trajectory, or response to current emerging therapies. In alignment with our study, Mszar et al. suggested that the investigation of patients carrying IBD pathogenic gene variants is pivotal for addressing the underdiagnosed rate of FH in specific geographical territories, while identifying CV risks in these genetically self-contained communities [15]. Similar findings were also described within the Indian population, showing the high prevalence of FH and unique genetic architecture caused by consanguineous marriage practices as well as multiple endogamous groups [16,17].

This PoC study has limitations. First, with a total of 105 participants, the sample size is small, although it offered sufficient power to highlight significant differences in lipid parameters. This said, it is worth mentioning that the pathogenic variants used in this PoC study were limited to those with a proven and published IBD origin. The establishment of a France–Canada transatlantic collaboration improves the identification of FH patients carrying proven IBD pathogenic variants in both countries, thus progressively contributing to the increase in the sample size. In addition, other FH-causing variants are currently being assessed for a common origin (founder effect). Such variants, if proven IBD, will be added in the next phases of the study, further increasing the patient pool. Second, this study specifically and only targets HeFH. We are currently integrating HoFH in order to maximize the advantages conferred by IBD pathogenic variants across various founder populations and multiple countries. Third, although available for several patients in secondary prevention, coronary anatomy data were not included in this PoC study. This will be the subject of a dedicated manuscript in a larger sample of age- and gender-matched FH patients with ASCVD.

In the next few years, planned analyses include exome sequencing, DNA methylation studies, as well as comprehensive endophenotypic, clinical, and environmental investigations in a larger sample of IBD patients. These efforts aim to better document modifiers of FH, uncover potential new targets for preventing ASCVD, and identify factors that modulate responses to emerging therapies. Integrating these diverse approaches within the clinical pathway will enhance our understanding of FH and help improve treatment strategies for affected populations on different continents. Populations from other countries, such as Lebanon, the USA, the UK, Turkey, India, and South Africa, will be involved in the next steps as these communities also share FH-causing IBD variants [18,19,20]. These populations importantly differ in terms of life habits and access to accurate diagnosis and treatment. Access issues and quality of life will also be assessed through this project as part of the SMASH initiative (System and Molecular Approaches of Severe Dyslipidemias) (accessed on 23 September 2024, www.smash-access.org).

## Figures and Tables

**Table 1:** Characteristics of HeFH patients from France and French Canada.

Characteristics (% or Median (IQR))	France (n = 30)	French Canada (n = 75)	*p*-Value
Men (%)	57	52	NS
Age (years)	53.0 (35.8–70.5)	61.0 (44.0–74.0)	NS
ApoE2 carriers (%)	4	14	NS
ApoE4 carriers (%)	17	29	NS
Diabetes (%)	3	7	NS
Obesity (%)	11	19	NS
Hypertension (%)	17	22	NS
Current smokers (%)	31	22	NS
Statin intolerance (%)	4	19	NS
ASCVD (%)	33	33	NS
Age at first event (years)	49.0 (39.8–56.0)	45.0 (39.5–54.5)	NS

NS = *p* > 0.05. Continuous variables are median (IQR). ApoE: apolipoprotein E, ASCVD: atherosclerotic cardiovascular disease, IQR: interquartile range.

**Table 2:** Gene variant-specific differences in untreated LDL-cholesterol concentration in France versus French Canada.

IBD Pathogenic Variants	France n (%)	LDL-c (mmol/L) Mean ± SD	French Canada n (%)	LDL-c (mmol/L) Mean ± SD
All variants	30 (100)	7.94 ± 1.86	75 (100)	6.93 ± 1.78
p.(Trp87Gly)	10 (33.3)	7.15 ± 2.10	38 (50.7)	6.64 ±1.66
p.(Cys667Tyr)	2 (6.7)	8.34 ± 0.71	9 (12.0)	6.63 ± 1.28
p.(Glu228Lys)	11 (36.7)	7.72 ± 1.81	15 (20.0)	7.50 ± 2.14
p.(Arg350*)	7 (23.3)	8.49 ± 2.00	13 (17.3)	7.05 ± 2.04

p.(Trp87Gly): c.259T>G, rs121908025. p.(Cys667Tyr): c.2000G>A, rs28942083. p.(Glu228Lys): c.682G>A, rs121908029. p.(Arg350*): c.1048C>T, rs769737896.

## Data Availability

The datasets presented in this article are not readily available because the data are part of an ongoing study. Genealogical data are not available due to privacy and ethical reasons. Requests concerning access to the datasets should be directed to the corresponding author.
